# Laparoscopic Treatment of a Symptomatic Young Woman With Median Arcuate Ligament Syndrome

**DOI:** 10.7759/cureus.61989

**Published:** 2024-06-09

**Authors:** Zoi Nitsa, Prodromos Kanavidis, Natasha Hasemaki, Athanasios Katsargyris, Alexandros Charalabopoulos

**Affiliations:** 1 First Department of Surgery, Laiko General Hospital of Athens, Athens, GRC; 2 Second Department of Vascular Surgery, Laiko General Hospital of Athens, Athens, GRC; 3 First Department of Surgery, National and Kapodistrian University of Athens, Athens, GRC

**Keywords:** atypical mals, dunbar syndrome, celiac axis syndrome, celiac artery compression syndrome, median arcuate ligament syndrome

## Abstract

Median arcuate ligament syndrome (MALS), also known as Dunbar syndrome, celiac axis syndrome, or celiac artery compression syndrome, is caused by a band of tissue called the median arcuate ligament that compresses the celiac artery and sometimes the celiac plexus too. MALS does not always cause symptoms, but when symptoms occur, surgery is the treatment of choice. This case report focuses on the case of a 27-year-old woman presenting with postprandial episodes of abdominal pain and vomiting accompanied by loss of weight, which was found to be MALS.

## Introduction

Median arcuate ligament syndrome (MALS) can cause symptoms like abdominal pain, nausea, vomiting after a meal, and diarrhea that can lead to fear of eating and significant weight loss. Surgical treatment consists of decompression of the celiac artery by dissecting the abnormal median arcuate ligament, which passes over the origin of the celiac trunk instead of above it [1]. Surgical treatment does not always lead to symptom resolution, and some of the patients are still having the same symptoms as before [2].

## Case presentation

**Figure 1:**
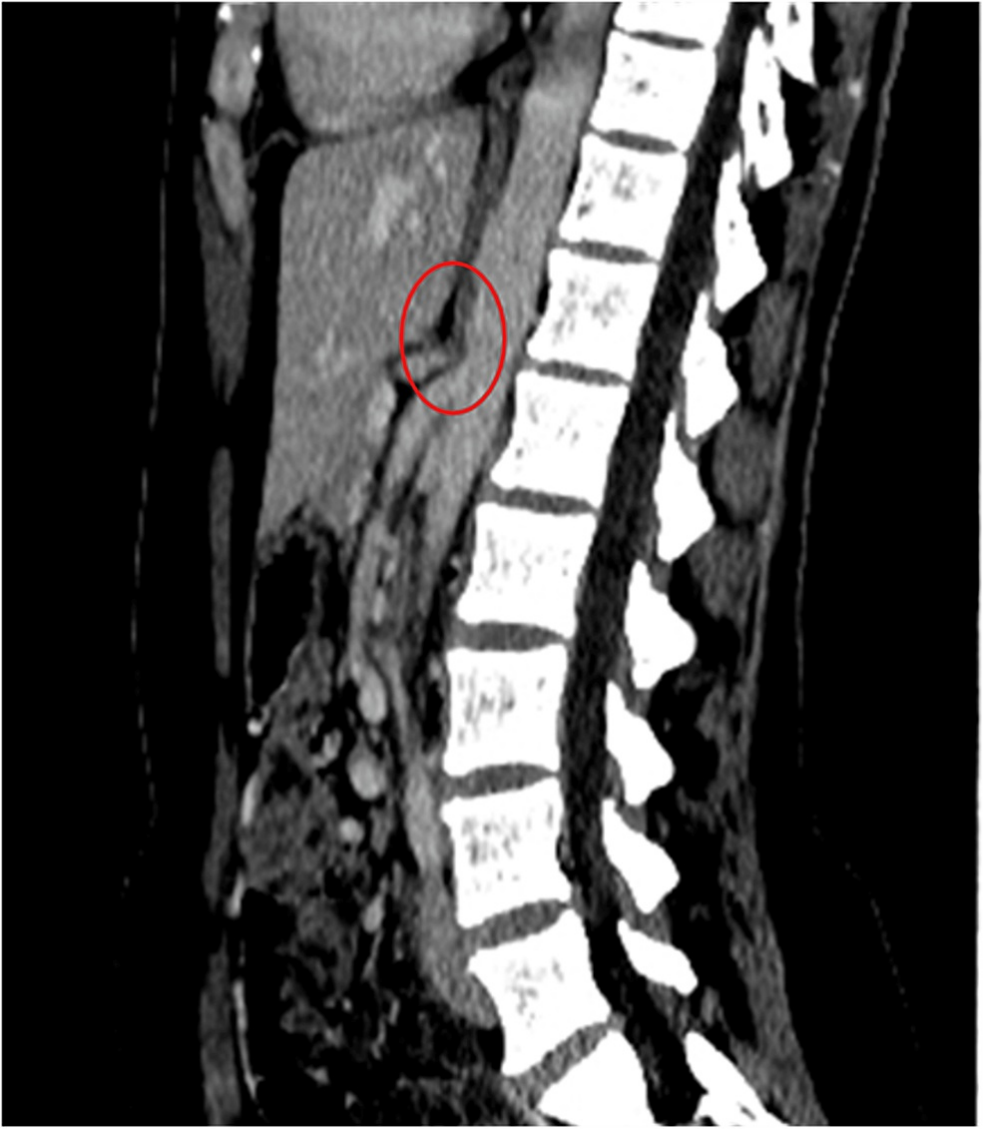
CT (sagittal plane) showing a hook-like sign CT: computed tomography

**Figure 2:**
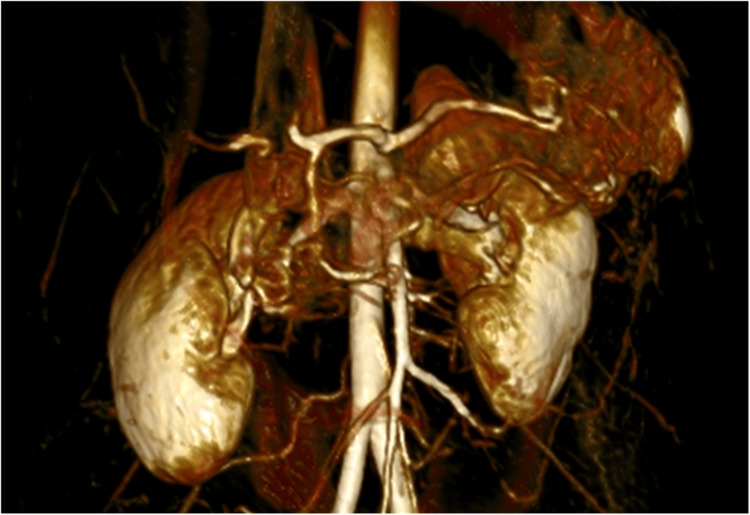
MRA 3D reconstruction of the celiac trunk MRA: magnetic resonance angiography, 3D: three dimensional

A 27-year-old woman presented with a nine-month history of weight loss, postprandial abdominal pain, and episodes of vomiting and diarrhea. The blood tests, ultrasound study, and endoscopy did not show any abnormalities. The patient underwent CT with intravenous contrast fluid infusion (Figure 1), later MR angiography (MRA) (Figure 2), and a triplex ultrasound study of the aorta and visceral branches, the results of which indicated MALS, with an expiratory peak celiac artery flow of 300 cm/s and almost complete flow obstruction at intense expiration.

**Figure 3:**
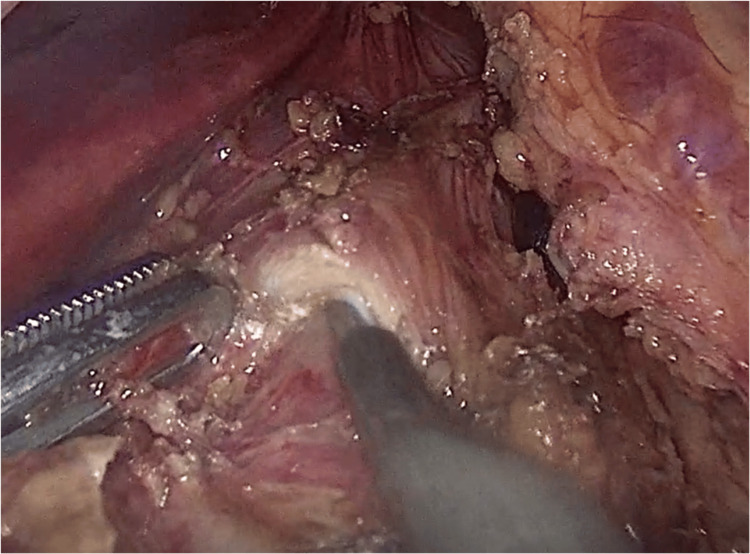
Release of the celiac trunk from the abnormal median arcuate ligament

**Figure 4:**
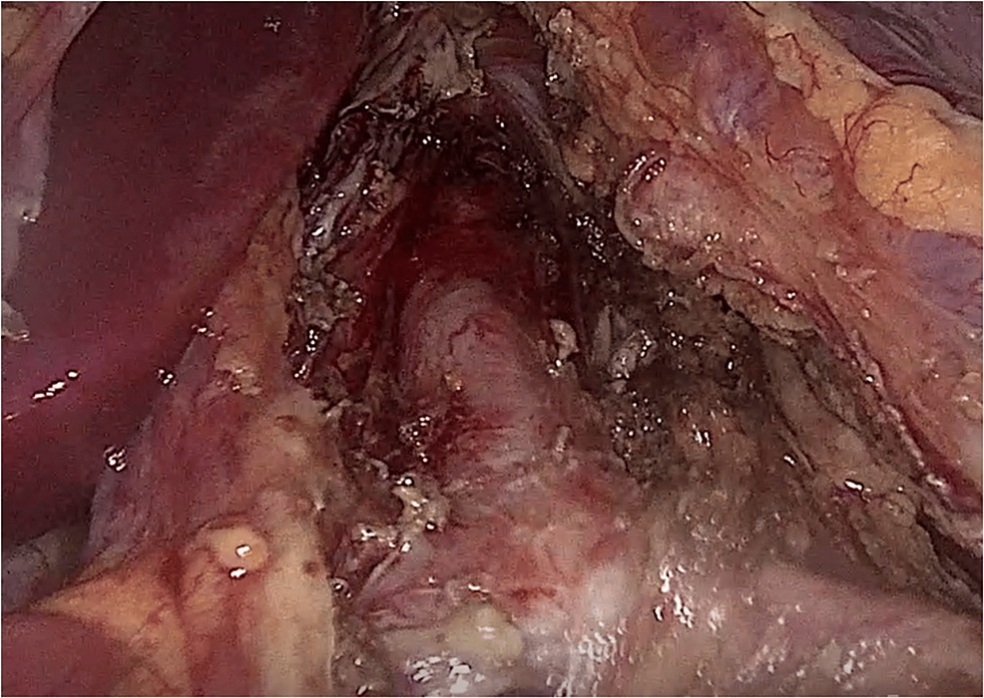
Released celiac trunk

The patient was treated laparoscopically by surgical release of the celiac artery and plexus from the pressure of the median arcuate ligament and lymphatic tissue (Figures 3-4). The operation lasted 35 minutes, and the blood loss was estimated to be 30 ml. On postoperative day 1, the patient was started on a clear liquid diet without postprandial abdominal pain or vomiting/diarrhea. The patient was discharged three days later, after progressing to a free diet and having an uncomplicated postoperative course.

The patient remained asymptomatic, tolerated food intake adequately, and gained 4 kg of weight in the first three months. She underwent a triplex study of the aorta and visceral branches with satisfactory results and improvement of the peak expiratory celiac artery flow (255 cm/s).

## Discussion

The MAL syndrome includes a set of clinical signs and symptoms resulting from compression of the celiac trunk and plexus by the median arcuate ligament. MALS, however, does not always cause symptoms. This has caused controversy because stenosis in the celiac artery will not always manifest postprandial ischemia in the gastric mucosa due to the development of collateral vasculature that provides sufficient blood flow so as not to cause symptoms and pain. Another possible factor is the compression of the celiac ganglion, which leads to the stimulation of sympathetic pain fibers and fibers of vasoconstriction [3]. The symptoms of MALS may vary, including postprandial abdominal pain, vomiting, diarrhea, and weight loss. Due to the similar symptoms with other more common diseases, the diagnosis of MALS is usually made by exclusion [4]. The diagnosis of MALS is made by duplex ultrasound, which diagnoses the stenosis of the celiac trunk by the expiratory peak systolic flow velocities (sometimes more than 350 cm/s) and deflection angle (>50°). In addition, CT angiography is an important diagnostic tool because it can assess the whole abdominal cavity, and in cases of MALS, it can show a hook-like sign of the celiac trunk that differentiates from atherosclerosis. MRA and digital subtraction angiography are alternative choices [5]. Recent studies have shown that a reverse flow in the gastro-duodenal artery, measured either by ultrasound or MRA, can be a marker of equal or better sensitivity than celiac artery stenosis, with reverse flow being observed from 35% stenosis and greater [3]. This could lead to a better selection of patients, a better correlation with symptoms, and possibly better outcomes. Surgical treatment is currently recommended only for symptomatic patients with 50% stenosis of the celiac artery [6]. The treatment consists of decompression of the celiac trunk, traditionally by an open approach. Recently, however, minimally invasive approaches such as laparoscopic or robotic are more often used, with similar or better results [7,8]. Data from cohorts of laparoscopic MALS decompression seems to show fewer postoperative complications, pain, and a shorter length of stay compared with the open approach [9]. Almost 70% of operated patients experience relief of symptoms [2]. With regards to the patients who continue to have unresolved symptoms, vascular bypass or angioplasty is the next step in treatment, while some patients benefit from celiac ganglion neurolysis or ablation [10].

## Conclusions

The MAL syndrome has a direct impact on the patient’s life, while the exact incidence of the disease is unknown. The exact pathophysiologic mechanism is not known; however, due to the low incidence of the disease, there are no guidelines for treatment. The laparoscopic decompression of the celiac artery in specialized centers seems to have promising results, with prompt relief of symptoms as well as immediate recovery and a quick return to daily activities, avoiding the complications of open surgery.
